# Utility of shock index to predict mortality risk in decompensated heart failure

**DOI:** 10.4102/jcmsa.v3i1.193

**Published:** 2025-07-24

**Authors:** Amanda Naidoo-Pillay, Suma Rajan, Andreas Engelbrecht

**Affiliations:** 1 Division of Emergency Medicine, Faculty of Health Sciences, University of Pretoria, Pretoria, South Africa; 2 Division of Emergency Medicine, Faculty of Health Sciences, Steve Biko Academic Hospital, Pretoria, South Africa; 3 Division of Emergency Medicine, Faculty of Health Sciences, Kalafong Provincial Tertiary Hospital, Pretoria, South Africa; 4 Department of Emergency Medicine, Faculty of Health Sciences, Letterkenny University Hospital, Galway, Ireland

**Keywords:** shock index, symptomatic heart failure, modified shock index, reverse shock index, mortality, inotropes

## Abstract

**Background:**

Decompensated heart failure (HF) is a serious condition that requires rapid evaluation and treatment. Shock index (SI) is a bedside predictor tool used to identify risk of mortality and morbidity.

**Methods:**

This multicentre retrospective descriptive study evaluated SI, modified shock index (MSI) and reverse shock index (RSI) as predictors of mortality in decompensated HF. Emergency department records for a 6-month period were analysed. The primary outcome was to identify if SI > 0.9 could predict mortality during hospital admission. Secondary outcomes included SI > 0.9, MSI > 0.93 and RSI < 1 for prediction of 72-h mortality, use of non-invasive positive pressure ventilation, inotropic, vasopressor or nitroglycerine infusions and need for endotracheal intubation.

**Results:**

Overall mortality during hospital admission was 4.3%, impacting internal validity. For inotrope use, SI > 0.9 at 12 h produced an odds ratio (OR) of 12.33 (confidence interval [CI] 2.03–74.89; *P* = 0.006). Modified shock index > 0.93 suggested potential for mortality and critical intervention prediction but lacked statistical significance. Reverse shock index < 1.0 at 0 h indicated ORs of 8.89 (CI 1.11–70.98) for in-hospital mortality and 9.88 (CI 1.70–57.27) for inotrope use.

**Conclusion:**

SI > 0.9 at 12 h predicted an increased need for inotropes. Reverse shock index demonstrates significant predictive value for mortality and critical interventions in decompensated HF. Reverse shock index appears to be the most effective index in predicting both mortality and need for critical interventions, outperforming SI and MSI. Further prospective studies are needed to validate these findings.

**Contribution:**

This research will assist with early triage of decompensated HF patients to appropriate dispositions and guide inexperienced clinicians to identify high-risk patients.

## Introduction

Decompensated heart failure (HF), that is, American Heart Association stage C to D heart failure^1^ (acute or acute on chronic), is a rising global epidemic and is one of the leading causes of hospital admissions in the geriatric population.^2^ Decompensated HF is a disease of high mortality and morbidity. Unfortunately, timely disposition to the appropriate level of care remains challenging. More than one-third of patients have had severe enough disease to require admission, demise or readmission within 3 months of the first consultation.^3,4^

Early recognition of symptoms in low-risk patients by enhancing the primary screening process; timely initiation of appropriate treatment and effective follow-up care are critical for improving patient outcomes. These strategies can also substantially reduce costs to the healthcare (HC) system and potentially mitigate hospitalisation rates.^5,6^ Prompt identification and assessment of risk profile allow for rapid recognition of individuals requiring emergency interventions, potentially decreasing morbidity and mortality.^7,8^ Augmenting the current triaging system with additional predictive indices will facilitate this.

Effective triage in an emergency department (ED) forms an imperative part of the approach to patient management and the prioritisation of patients. In the context of South African (SA) public HC, most EDs are overwhelmed with patient numbers far exceeding the available resources. With our ageing population, socio-economic strain^9^ and increasing lifestyle, and communicable and non-communicable diseases,^10^ decompensated HF is a common presentation to the ED. Early risk stratification can lead to appropriate patient disposition and outcomes of the disease can be improved. In resource-limited settings, young inexperienced clinicians are often the first point of contact.

In such instances, effective risk stratifying tools can be utilised promptly to identify patients who are at high risk of mortality and ensure early consultation with senior colleagues; up triaging to the appropriate level of care as indicated. This will aid in facilitating ethical distribution of scarce resources.

The shock index (SI) was first described and used in 1967 to assess patients with haemorrhagic shock.^11^ The SI, by definition, is an index calculated by dividing the heart rate (HR) by the systolic blood pressure (BP) of a patient. It has been found, in a number of patient cohorts, to be a better aid when identifying ill patients at risk of mortality when compared to reviewing the HR and the BP as individual parameters.^12,13,14,15^ The basic concept of the SI has been modified over time to the modified shock index (MSI), which is calculated by dividing HR by the mean arterial pressure. The MSI incorporates the diastolic BP into the calculation, which in certain patient cohorts has been shown to be more sensitive in predicting the risk of deterioration.^16^ In a retrospective observational study an MSI of > 0.93 was able to predict the need for inotropic therapy, invasive ventilation and 6-month mortality in a cohort of patients with ST-elevation myocardial infarctions.^17^

Further evaluation of the SI led to the development of another index called the reverse shock index (RSI). This index has shown value in predicting haemodynamic instability in trauma patients with a cut-off value of > 1. The ratio is inverse to that used in SI calculations and is calculated by dividing systolic BP by HR.^18^

The SI, MSI and RSI are inexpensive bedside tools and provide important information for chronically and critically ill patients. These scores do not require additional laboratory results in order to complete mortality risk prediction and can be performed when the patient arrives at the ED for triage. Scores and scales that help to expedite appropriate treatment will improve triage efficiency and outcomes. The SI was investigated for use in acute HF by El-Menyar et al.^19^ and it was found that an SI cut-off > 0.9 provided statistically significant information regarding mortality and cardiogenic shock prediction in the local population.

Our study aims to appraise the use of SI (MSI and/or RSI) to identify patients with decompensated HF who are at risk of acute deterioration, including mortality or the need for critical intervention in our local patient cohort. The study aims to identify if SI (MSI and/or RSI) can be utilised in conjunction with the current triaging tools employed by various facilities to better identify a patient at high risk of mortality and expedite their critical interventions and appropriate level of disposition. Furthermore, we also aim to compare the prognostic value of SI to MSI and RSI in HF as it has not been explored in the SA and broader African setting.

## Research methods and design

### Study design and setting

This multicentre retrospective descriptive study was conducted at one central academic hospital and one provincial tertiary hospital in Gauteng, South Africa between 01 January and 30 June, 2019. The data were collected retrospectively, from paper-based ED records of patients with decompensated HF. Records were updated by the nurses at the admission area and/or triage area and subsequently the administration clerk captured it. A symptom log with ED presentation diagnosis was recorded (diagnosis by ED doctor after assessment). The International Classification of Diseases, Tenth Revision (ICD 10) codes were captured by the administration team post admission diagnosis. The primary objective of the study was to determine the effectiveness of SI of ≥ 0.9 in predicting in-hospital mortality in decompensated HF patients. The secondary outcomes included the predictive value of an MSI ≥ 0.93 and/or RSI < 1.0 in comparison to SI to predict mortality during admission. Other secondary outcomes were to assess if an SI of ≥ 0.9, an MSI of ≥ 0.93 and/or an RSI < 1 were able to predict 72-h mortality; and could identify which patients with a high risk of mortality required one or more of the following critical interventions: inotropes, vasopressors, nitroglycerine intravenous infusion, endotracheal intubation and/or non-invasive positive pressure ventilation (NIPPV).

### Study population and sampling strategy

A sample of 93 patients (exceeding the calculated minimum of 85 for 95% confidence intervals [CI] with expected sensitivity and specificity of 0.9 and 0.8, respectively) was achieved.

The study included adult patients (18–85 years) presenting to the ED with a clinical diagnosis of decompensated HF (high output, HF with reduced ejection fraction (EF) or HF with preserved EF) made by the ED doctor, who then referred the patient for admission by internal medicine. The EDs were staffed by medical interns, community service medical officers, medical officers, emergency medicine registrars and emergency medicine specialists (all junior doctors perform clinical duties under the supervision of senior staff). Ideally, the clinical diagnosis of HF was supported by either an abnormal pro-brain natriuretic peptide (Pro-BNP)^20^ (≥ 100 picogram/mL) and/or an echocardiogram (ECHO) diagnosis of HF if available. Echocardiograms were performed by radiology at the tertiary facility and by either cardiology or radiology at the central facility. Patients with acute kidney injury (AKI) on admission, who had a normalised serum creatinine within ± 7 days of admission on a repeat renal function blood panel were admitted into the study. Given the resource limitations, the clinical diagnosis of decompensated HF was prioritised when a Pro-BNP and ECHO were unavailable.

The following exclusion criteria were applied: out-of-hospital cardiac arrest; renal failure (AKI – KDIGO AKI >/= stage 1; chronic kidney disease [CKD] – KDIGO CKD >/=2)^21,22,23^; fulminant liver failure^24^; septic shock^25^; other forms of shock (obstructive, distributive and hypovolaemic shock); and incomplete and/or missing files.

### Data collection

All patients recorded in the handwritten ED register with an initial diagnosis of HF for the study period were screened. The clinical records were used to collect the relevant demographic data; HR and BP values (as close as possible to the time intervals); patient comorbidities and specific treatments that may affect the indices, for example, beta-blocker (BB), interventions applied, mortality occurrence or length of stay. The SI, MSI and RSI were calculated retrospectively for included patients using the HR and BP. Cut-off values for the indices were based on established literature.^17,18,19^ The data were captured on an excel spreadsheet and stored in soft and hard copy formats, in password-protected and an access-controlled filing room, respectively.

### Data analysis

A probability sampling method was utilised and a stratified sampling tool was used. Linear correlations between various clinical variables were evaluated to identify any patient factors that may correlate to an elevated SI, MSI or RSI. Normally distributed data were reported as a mean with ± standard deviation (s.d.) and abnormally distributed data were reported as a median and an associated interquartile range.

The data were analysed using IBM SPSS statistics version 30. Descriptive statistics present the patients’ demographics and clinical profiles and are shown in frequencies (*n*) and percentages (%). Univariable binary logistic regression models were utilised to calculate odds ratios (OR) for the association between various shock indices and mortality or intervention requirements. Statistical significance was set at *p* < 0.05. Associations between SI categorical levels and mortality risk were tested in 2-way contingency tables and using Fisher’s exact test. The Mann–Whitney (U) test was used for the same purpose when SI was analysed as a continuous variable. The reliability of the various types and levels of SI as a predictive tool for mortality was tested in two-by-two cross-tabulations to determine its diagnostic sensitivity and specificity. The receiver operating characteristic curves (ROC) were plotted to visualise the predictive performance of SI, MSI and RSI.

### Ethical considerations

The study adhered to the ethical guidelines of the Declaration of Helsinki (1975, revised 2013) and the ethical principles of each participating institution. Ethical clearance to conduct this study was obtained from the University of Pretoria Faculty of Health Sciences Research Ethics Committee (No. 10/2020).

The Chief Executive Officer of each hospital granted permission to access the files to collect data and the study was also registered on the National Health Research Database. Patients were allocated sequential numbers instead of personal identifiers to ensure anonymity. Data collection and analysis were carried out in accordance with the *Protection of Personal Information Act* (POPI) of South Africa. No patient identifiers have been stated or alluded to in this article.

## Results

**FIGURE 1:**
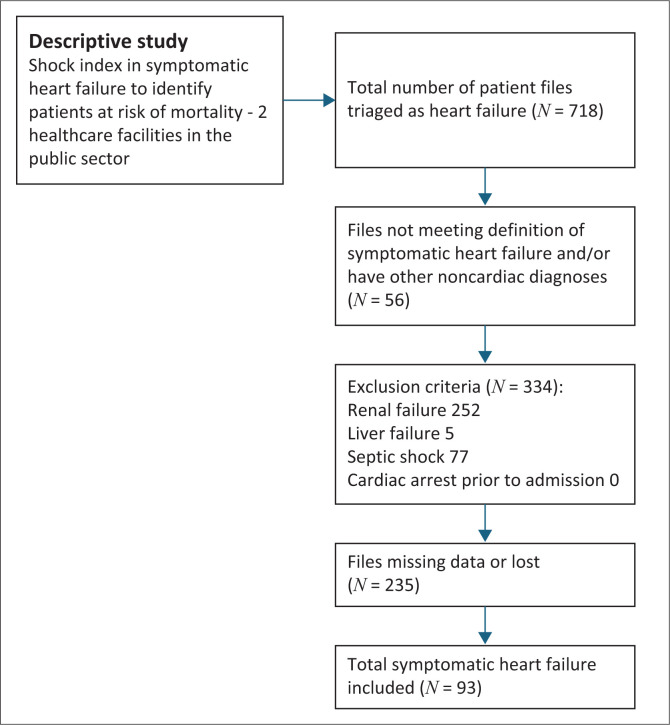
Flowchart of patients admitted into study.

The study screened 718 patient files of which 93 patients met the inclusion criteria. Figure 1 demonstrates the flowchart of included patients as well as additional exclusions noted during the recruitment process. Of the total patients included, 32.3% were identified at the provincial tertiary hospital and 67.7% at the central academic hospital. The mean age of the 93 patients was 55 (± s.d. 14.4) years. Female patients were 50 (53.8%) and male patients were 43 (46.2%). Because of the paper-based record system many files were either lost or had missing notes (e.g. vital signs charts not found or treatment charts not found, etc.).

Table 1 demonstrates the patient demographics and baseline characteristics of those included in this study. Sixty per cent of patients were hypertensive and of those patients, 29.03% were on BB therapy, 13.98% were on calcium channel blockers (CCB), 32.26% were on angiotensin-converting enzyme inhibitors (ACEI) and 51.61% were on diuretic therapy. Sixty-four per cent of patients had ECHOs, of which 45% had left ventricular ejection fractions of < 40%.

**TABLE 1:** Patients’ demographics (*N* = 93).

Patient demographics and baseline characteristics	*n*	% of total
**Gender**		
Male	43	46.24
Female	50	53.76
**Age (years)** †		
18–30	4	4.30
31–40	14	15.05
41–50	20	21.51
51–60	17	18.28
61–70	25	26.88
71–80	10	10.75
> 80	3	3.23
**Race**		
African people	60	64.52
Caucasian people	27	29.03
Asian people	3	3.23
Mixed race people	3	3.23
**Employment status**		
Unemployed	39	41.94
Pensioner	34	36.56
Employed	18	19.35
Student	2	2.15
**Comorbidities**		
Hypertension	56	60.22
Pre-existing cardiac disease‡	38	40.86
Smoker	30	32.26
Chronic obstructive airway disease	20	21.51
Diabetes	18	19.35
Dyslipidaemia	11	11.83
Human immunodeficiency virus	8	8.60
Asthma	5	5.38
Epilepsy	1	1.08
**Specific therapies**		
Diuretic therapy	48	51.61
ACEI	30	32.26
BB therapy	27	29.03
CCB therapy	13	13.98
**New York Heart Association for Dyspnoea**		
Grade 1	1	1.08
Grade 2	9	9.68
Grade 3	16	17.20
Grade 4	30	32.26
Undefined	37	39.78

ACEI, angiotensin-converting enzyme inhibitor; BB, beta-blocker; CCB, calcium channel blocker; s.d., standard deviation.

† , Mean 55 years (±s.d.^ 14.4);

‡ , Cardiomyopathy; valve lesions; pulmonary hypertension; dysrhythmias; previous bypass; previous myocardial infarction; previous HF.

### Mortality

During the hospital admission periods, four mortalities accounted for 4.3% of the sample; two deaths occurred within the first 72 h. The OR for SI and MSI across the various time intervals for the primary outcome did not indicate statistical significance. However, the RSI demonstrated a significant association with mortality with an OR = 8.89 (CI 1.11–70.98; *p* = 0.04) at 0 h, suggesting a statistically significant predictor of mortality during admission. Further studies in larger cohorts are required. Table 2 demonstrates the results of the primary outcome (i.e. SI and mortality during admission) with regard to ORs while Table 3 demonstrates the secondary outcomes of the study.

**TABLE 2:** Odds ratios for primary outcome.

Mortality prediction during hospital admission	Odds ratio	Confidence interval	*p*-value
SI 0 h	3.94	0.52–29.95	0.18
SI 12 h	0.64	0.03–12.92	0.77
SI 24 h	3.94	0.24–66.16	0.34
SI 72 h	4.24	0.25–71.18	0.32

SI, Shock index.

**TABLE 3:** Odds ratios for secondary outcomes.

Predictive indices	Odds ratio	Confidence interval	*p*-value
**SI 0 h**			
72-h mortality	3.79	0.23–63.42	0.35
Inotrope	4.12	0.76–22.22	0.10
Vasopressor	0.70	0.03–15.12	0.82
NIPPV	1.05	0.20–5.49	0.96
Endotracheal intubation	0.70	0.03–15.12	0.82
**SI 12 h**			
72-h mortality	1.53	0.06–39.13	0.80
Inotrope	12.33	2.03–74.89	< 0.01
Vasopressor	5.00	0.30–84.45	0.26
NIPPV	1.41	0.26–7.50	0.69
Endotracheal intubation	5.00	0.30–84.45	0.26
**SI 24 h**			
72-h mortality	n/a	n/a	n/a
Inotrope	4.31	0.80–23.38	0.09
Vasopressor	0.72	0.03–15.69	0.84
NIPPV	0.44	0.05–3.79	0.46
Endotracheal intubation	0.72	0.03–15.70	0.84
**SI 72 h**			
72-h mortality	n/a	n/a	n/a
Inotrope	4.67	0.86–25.41	0.07
Vasopressor	4.24	0.25–71.18	0.32
NIPPV	0.48	0.06–4.09	0.50
Endotracheal intubation	0.77	0.04–16.80	0.87
**MSI 0 h**			
Mortality during admission	0.85	0.12–6.34	0.88
72-h mortality	0.86	0.05–14.13	0.91
Inotrope	12.71	0.69–232.51	0.09
Vasopressor	0.86	0.05–14.13	0.91
NIPPV	1.82	0.43–7.76	0.42
Endotracheal intubation	0.86	0.05–14.13	0.91
**MSI 12 h**			
Mortality during admission	4.78	0.24–95.25	0.31
72 h mortality	1.97	0.08–49.77	0.68
Inotrope	9.40	0.51–172.11	0.13
Vasopressor	3.35	0.16–71.79	0.44
NIPPV	0.78	0.20–3.14	0.73
Endotracheal intubation	3.35	0.16–71.79	0.44
**MSI 24 h**			
Mortality during admission	0.65	0.04–10.70	0.76
72-h mortality	n/a	n/a	n/a
Inotrope	9.59	0.52–175.63	0.13
Vasopressor	3.41	0.16–73.15	0.43
NIPPV	1.35	0.31–5.77	0.69
Endotracheal intubation	3.41	0.16–73.15	0.43
**MSI 72 h**			
Mortality during admission	5.59	0.26–119.72	0.27
72-h mortality	n/a	n/a	n/a
Inotrope	5.90	0.66–52.64	0.11
Vasopressor	5.59	0.26–119.72	0.27
NIPPV	0.50	0.12–2.14	0.35
Endotracheal intubation	5.59	0.26–119.72	0.27
**RSI 0 h**			
Mortality during admission	8.89	1.11–70.98	0.04
72-h mortality	8.10	0.47–139.84	0.15
Inotrope	9.88	1.70–57.27	0.01
Vasopressor	1.40	0.06–31.04	0.83
NIPPV	0.93	0.10–8.19	0.94
Endotracheal intubation	1.40	0.06–31.04	0.83
**RSI 12 h**			
Mortality during admission	1.21	0.06–25.27	0.90
72-h mortality	2.89	0.11–76.19	0.52
Inotrope	13.33	2.20–80.87	< 0.01
Vasopressor	10.25	0.58–179.94	0.11
NIPPV	1.17	0.13–10.60	0.89
Endotracheal intubation	1.72	0.08–38.47	0.73
**RSI 24 h**			
Mortality during admission	7.90	0.46–136.42	0.16
72-h mortality	n/a	n/a	n/a
Inotrope	4.22	0.68–26.39	0.12
Vasopressor	1.37	0.06–30.27	0.84
NIPPV	0.90	0.10–7.97	0.92
Endotracheal intubation	1.37	0.06–30.27	0.84
**RSI 72 h**			
Mortality during admission	13.83	0.77–249.67	0.08
72-h mortality	n/a	n/a	n/a
Inotrope	2.63	0.26–26.32	0.41
Vasopressor	13.83	0.77–249.67	0.08
NIPPV	0.53	0.03–10.03	0.67
Endotracheal intubation	2.20	0.10–50.18	0.62

SI, shock index; MSI, modified shock index; RSI, reverse shock index; NIPPV, non-invasive positive pressure ventilation; n/a, not applicable.

Although RSI did not demonstrate a statistically significant association with predicting mortality at 72 h, there appears to be an association (OR of 13.83; CI 0.77–249.67; *p* = 0.08). The wide CI and non-significant *p*-value suggest a need for further study in larger cohorts (see Table 3).

The prediction values for mortality at 72 h were as follows: SI > 0.9 had a sensitivity of 50% (1.258% to 98.742%) and specificity of 80.68% (70.881% to 88.325%).

Modified shock index > 0.93 had a sensitivity of 100% (15.811% to 100%) and specificity of 52.81% (41.937% to 63.489%). This MSI analysis had statistical significance with *p* = 0.011. Reverse shock index < 1 had a sensitivity of 50% (1.258% to 98.742%) and a specificity of 93.26% (85.902% to 97.486%). Table 4 compares the sensitivities, specificities, *p*-values and CIs for several parameters for SI, MSI and RSI.

**TABLE 4:** Sensitivities, specificities, *p*-values, and confidence intervals for several parameters for shock index, modified shock index and reverse shock index.

Predictive tool at varying time intervals	Sensitivity (%)	Specificity (%)	*p*-value	Confidence interval
**SI 0 h**				
Mortality during admission	50.00	79.78	0.33	6.76–93.24
72-h mortality	50.00	79.12	0.50	1.26–98.74
Inotrope	50.00	80.46	0.23	11.81–88.19
**MSI 0 h**				
Mortality during admission	50.00	46.07	0.90	6.76–93.24
72-h mortality	50.00	46.15	0.93	1.26–98.74
Inotrope	100.00	49.43	< 0.001	54.07–100.00
**RSI 0 h**				
Mortality during admission	50.00	89.89	0.21	6.76–93.24
72-h mortality	50.00	89.01	0.38	1.26–98.74
Inotrope	50.00	90.81	0.12	11.81–88.19
**SI 12 h**				
Mortality during admission	0.00	82.02	0.53	0.00–70.76
72-h mortality	0.00	81.61	0.72	0.00–97.50
Inotrope	66.67	86.05	0.02	22.28–95.67
NIPPV	22.22	83.13	0.80	2.81–60.00
**MSI 12 h**				
Mortality during admission	100.00	40.45	0.06	29.24–100.00
72-h mortality	100.00	39.56	0.27	2.50–100.00
Inotrope	100.00	41.86	< 0.01	54.07–100.00
NIPPV	55.56	38.55	0.77	21.20–86.30
**RSI 12 h**				
Mortality during admission	0.00	89.89	0.74	0.00–70.76
72-h mortality	0.00	90.11	0.85	0.00–97.50
Inotrope	50.00	93.02	0.10	11.81–88.19
NIPPV	11.11	90.36	0.94	0.28–48.25
**SI 24 h**				
Mortality during admission	50.00	79.78	0.49	1.26–98.74
72-h mortality	n/a	n/a	n/a	n/a
Inotrope	50.00	81.18	0.22	11.81–88.19
**MSI 24 h**				
Mortality during admission	50.00	39.33	0.80	1.26–98.74
72-h mortality	n/a	n/a	n/a	n/a
Inotrope	100.00	42.35	< 0.01	54.07–100.00
**RSI 24 h**				
Mortality during admission	50.00	88.76	0.38	1.26–98.74
72-h mortality	n/a	n/a	n/a	n/a
Inotrope	33.33	89.41	0.39	4.33–77.72
**SI 72 h**				
Mortality during admission	50.00	80.68	0.48	1.26–98.74
72-h mortality	n/a	n/a	n/a	n/a
Vasopressor	50.00	80.90	0.48	1.26–98.74
NIPPV	11.11	79.27	0.62	0.28–48.25
Endotracheal intubation	0.00	79.78	0.55	0.00–84.19
**MSI 72 h**				
Mortality during admission	100.00	52.81	0.01	15.81–100.00
72-h mortality	n/a	n/a	n/a	n/a
Vasopressor	100.00	52.81	0.01	15.81–100.00
NIPPV	33.33	50.00	0.40	7.49–70.07
Endotracheal intubation	100.00	52.81	0.01	15.81–100.00
**RSI 72 h**				
Mortality during admission	50.00	93.26	0.34	1.26–98.74
72-h mortality	n/a	n/a	n/a	n/a
Vasopressor	50.00	93.26	0.34	1.26–98.74
NIPPV	0.00	91.46	0.65	0.00–33.63
Endotracheal intubation	0.00	92.14	0.22	0.00–84.19

SI, shock index; MSI, modified shock index; RSI, reverse shock index; NIPPV, non-invasive positive pressure ventilation; n/a, not applicable.

**FIGURE 2:**
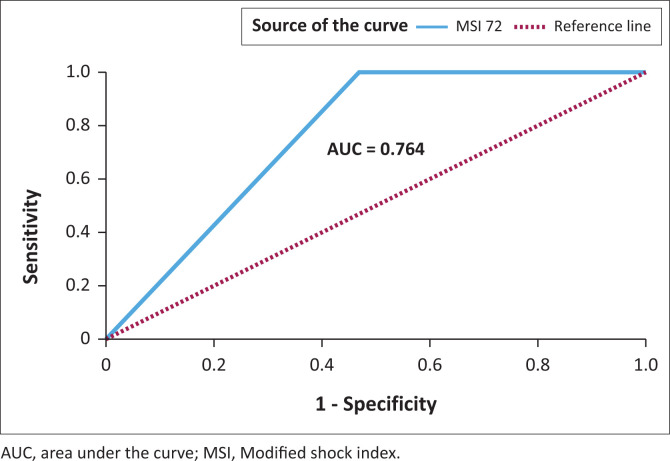
Receiver operating characteristic curve modified shock index at 72 h mortality prediction.

The ROC analysis for the area under the curve (AUC) for MSI at 72 h was 0.764 (CI 0.663 to 0.847) (See Figure 2).

### Secondary outcomes

Critical interventions utilised included NIPPV (9.68%), inotropic therapy (7.53%), endotracheal intubation (2.15%) and vasopressor therapy (2.15%); no patients were initiated on nitroglycerine infusions. Other therapies frequently used were facemask oxygen (31.18%), furosemide bolus doses (63.44%) and furosemide infusions (5.38%).

The SI showed a significant OR for predicting inotrope therapy at 12 h (OR = 12.33, *p* = 0.006).

The RSI was the most effective for predicting inotropic therapy requirements, with significant ORs at both 0 h (OR = 9.86, *p* = 0.01) and 12 h (OR = 13.33, *p* = 0.005) (see Table 3).

At 72 h the RSI < 1 had a specificity for NIPPV initiation of 91.46% while at 12 h the SI had an AUC for predicting inotrope support that was 0.764, which was statistically significant (*p* = 0.023).

The MSI at various time points showed statistically significant ROC and *p*-values; of note, the MSI at 72 h had a sensitivity of 100% to predict endotracheal intubation, specificity 52.81% and *p* = 0.011 (AUC 0.7) (see Table 4). This means that an MSI < 0.93 at 72 h is highly likely to rule out the need for endotracheal intubation; however, an MSI > 0.93 at 72 h does not necessarily rule in the requirement for intubation because of low specificity.

## Discussion

In this study, the SI, MSI and RSI were assessed as prediction tools for mortality for SA patients presenting with decompensated HF to the local ED. Of the total patients included in the study, 32.3% were from the tertiary hospital and 67.7% were from the central hospital. This was possibly because of the academic hospital having a cardiology department and having more referrals from peripheral facilities. We found that the OR for the use of SI to predict mortality in our study was not statistically significant. Shock index > 0.9 also had lower sensitivities for most of the parameters tested, which was consistent with observations in a trauma population.^26^ This highlights the need for further research on the applicability of vital sign-based scoring systems in decompensated HF patients within the SA context, particularly given the HF cohort studied by Costa et al.^27^ where they used study-derived cut-off values. Although the multivariate analysis of Costa et al.^27^ showed statistically significant *p*-values for SI and MSI in mortality prediction, the optimal cut-off values may need adjustment for our specific population.

Of note in our study, the MSI had a high sensitivity and statistically significant associations with mortality and the need for inotropic support, vasopressor therapy and endotracheal intubation at different time intervals. This is in keeping with the findings published by Liu et al.,^16^ who demonstrated the superior predictive ability of MSI for mortality in comparison to HR, BP and SI in a large cohort requiring intravenous fluid therapy.

Our study is the first to investigate RSI in HF patients within an African context. In our study, it was noticed that an RSI had statistically significant ORs across primary and secondary outcomes, with statistically significant prediction for inotropic therapy (at 0 h and 12 h) and mortality (at 0 h). These results partially align with Oh et al.^28^ who employed a modified RSI in reduced ejection fraction HF. Further investigations regarding the use of RSI to predict mortality and morbidity in larger HF cohorts are required to confirm these findings as it is not well studied currently.

In a study that was similar to ours, which was completed in 2019 by El-Menyar et al.,^24^ it was found that a cut-off value of SI > 0.9 was predictive of mortality and cardiogenic shock. The OR for SI at 12 h in our study to predict the need for inotropic support was statistically significant (*p* = 0.006; OR 12.33). This is in keeping with the findings of El-Menyar et al.^24^ for the high OR to predict cardiogenic shock (OR 9.26) in HF patients by use of the SI.

Other studies support the utility of SI, MSI and age-adjusted SI in earlier identification of acutely ill patients,^29,30^ with superior performance over BP alone in mortality prediction.^30^

The Persian registry for cardiovascular disease and HF found a strong association between SI and MSI and reduced survival at an SI > 0.66 and an MSI > 0.87.^31^ A recent meta-analysis corroborated these findings indicating a strong association between increased SI and MSI and in-hospital mortality.^32^ This suggests that further study regarding different SI and MSI cut-off points may be required in our specific cohort.

There is a large and ever-growing population of young patients who present with decompensated.^33^ An alarming finding in our study is that over 40% of the decompensated HF patients were less than 50 years old. This highlights the need for effective screening and management strategies at primary and secondary levels of HC to identify these patients early and prevent progression of the disease and deterioration. Further study is required in HF patients under 50 years.

It was observed during our review of current literature that standard care for decompensated or symptomatic HF according to American Heart Association guidelines,^34^ included the use of an angiotensin receptor or neprilysin inhibitor, as well as sodium-glucose cotransporter two inhibitors. However, because of resource limitations, these drugs are not widely available in the state sector. Further study is required regarding the implication of the drug therapies utilised and their impact on mortality and morbidity in the SA context.

### Limitations

This retrospective study using paper-based records over a 6-month period had several other limitations. In this study, we found a low mortality rate of 4.3% (4 patients), which impacted the statistical analysis of the primary outcome (mortality). Analysis in a larger cohort, over a longer data collection period, would have been valuable to limit type II errors in analysis and would have powered the study better. The paper-based record system with many lost and incomplete files reduced the sample size admitted into the study. Physician bias in diagnosis and intervention choices may have confounded the results. Physician preference for vasopressor and inotropic support may have also introduced variability in the HR values at 72 h, which would have impacted the indices at 72 h. Other limitations were inconsistent availability of Pro-BNP testing, renal function panels and regular ECHOs because of resource limitations. This impacted the objective diagnosis of HF and increased risk for physician biases. There was also a variation in vital signs measuring techniques and timing between facilities. Exclusion of patients with persistent renal dysfunction, dual burden disease (HF and renal dysfunction), will require further investigation as it formed a large cohort of patients.

## Conclusion

This study demonstrates the potential utility of readily available bedside tools – SI, MSI and RSI for risk stratification of patients with decompensated HF presenting to the ED.

Specifically, the SI measured 12 h post-presentation predicted the need for inotropic support. This requires further study in a larger cohort but the statistical significance in our study should create awareness of the implications of an elevated SI > 0.9 at 12 h in decompensated HF patients. Even though MSI and RSI were evaluated as secondary outcomes, they showed significant value in predicting mortality and the need for critical interventions within 72 h of presentation in this study.

Notably, RSI demonstrated the strongest ability to predict both mortality and inotropic support in our cohort, surpassing both SI and MSI. This may suggest that RSI, calculated upon arrival to the ED, could be a valuable tool for early risk stratification, thereby guiding resource allocation and improving patient outcomes. The relatively low mortality rate in our study underscores the need for further investigation to validate these findings in larger, prospective studies with standardised protocols and enhanced access to diagnostic testing.

## References

[1] American Heart Association, Inc. Classes and Stages of Heart Failure. [updated 2023 Jun 07; cited 2025 Mar 31]. Available from: https://www.heart.org/en/health-topics/heart-failure/what-is-heart-failure/classes-of-heart-failure

[2] Farmakis D, Lekakis J, Filippatos G. Acute heart failure: Epidemiology, risk factors, and prevention. Rev Esp Cardiol. 2015;68:245–248. 10.1016/j.recesp.2014.11.009 25659507

[3] Kurmani S, Squire I. Acute heart failure: Definition, classification and epidemiology. Curr Heart Fail Rep. 2017;14(5):385–392. 28785969 10.1007/s11897-017-0351-y PMC5597697

[4] Gheorghiade M, Abraham WT, Albert NM, et al. OPTIMIZE-HF Investigators and Coordinators. Systolic blood pressure at admission, clinical characteristics, and outcomes in patients hospitalized with acute heart failure. JAMA. 2006;296(18):2217–2226. 10.1001/jama.296.18.2217 17090768

[5] Collins SP, Lindsell CJ, Jenkins CA, et al. Risk stratification in acute heart failure: Rationale and design of the STRATIFY and DECIDE studies. Am Heart J. 2012;164(6):825–834. 10.1016/j.ahj.2012.07.033 23194482 PMC3511776

[6] Collins SP, Pang PS, Fonarow GC, Yancy CW, Bonow RO, Gheorghiade M. Is hospital admission for heart failure really necessary? The role of the ED and observation unit in preventing hospitalization and rehospitalization. J Am Coll Cardiol. 2013;61(2):121–126. 23273288 10.1016/j.jacc.2012.08.1022 PMC3535319

[7] Rame JE, Sheffield MA, Dries DL, et al. Outcomes after emergency department discharge with a primary diagnosis of heart failure. Am Heart J. 2001;142(4):714–719. 10.1067/mhj.2001.118473 11579364

[8] Chin MH, Goldman L. Correlates of major complications or death in patients admitted to the hospital with congestive heart failure. Arch Intern Med. 1996;156(16):1814–1820. 8790075

[9] Egbujie BA, Igumbor EU, Puoane T. A cross-sectional study of socioeconomic status and cardiovascular disease risk among participants in the Prospective Urban Rural Epidemiological (PURE) Study. S Afr Med J. 2016;106(9):900–906. 10.7196/SAMJ.2016.v106i9.10456 27601117

[10] Rising non-communicable diseases: A looming health crisis [homepage on the Internet]. Stats SA, Department: Statistics South Africa. C17 October 2023 [updated 2023 Oct 17; cited 2024 May 23]. Available from: https://www.statssa.gov.za/?p=16729

[11] Montoya KF, Charry JD, Calle-Toro JS, Núñez LR, Poveda G. Shock index as a mortality predictor in patients with acute polytrauma. J Acute Dis. 2015;4(3):202–204. 10.1016/j.joad.2015.04.006

[12] McCall SJ, Musgrave SD, Potter JF, et al. The shock index predicts acute mortality outcomes in stroke. Int J Cardiol. 2015;182:523–527. 10.1016/j.ijcard.2014.12.175 25661859

[13] Huang B, Yang Y, Zhu J, et al. Usefulness of the admission shock index for predicting short-term outcomes in patients with ST-segment elevation myocardial infarction. Am J Cardiol. 2014;114:1315–1321. 25201214 10.1016/j.amjcard.2014.07.062

[14] Sam A, Sanchez D, Gomez V, et al. The shock index and the simplified PESI for identification of low-risk patients with acute pulmonary embolism. Eur Respir J. 2011;37:762–766. 10.1183/09031936.00070110 20650994

[15] Rady MY, Smithline HA, Blake H, Nowak R, Rivers E. A comparison of shock index and conventional vital signs to identify acute, critical illness in the emergency department. Ann Emerg Med. 1994;24(4):685–690. 10.1016/S0196-0644(94)70279-9 8092595

[16] Liu Y, Liu J, Fang ZA, et al. Modified shock index and mortality rate of emergency patients. World J Emerg Med. 2012;3(2):114–117. 10.5847/wjem.j.issn.1920-8642.2012.02.006 25215048 PMC4129788

[17] Abreu G, Azevedo P, Braga CG, et al. Modified shock index: A bedside clinical index for assessment of ST-segment elevation myocardial infarction at presentation. Rev Port Cardiol (Engl Ed). 2018;37(6):481–488. 29807676 10.1016/j.repc.2017.07.018

[18] Chuang JF, Rau CS, Wu SC, et al. Use of the reverse shock index for identifying high-risk patients in a five-level triage system. Scand J Trauma Resusc Emerg Med. 2016;24:12. 10.1186/s13049-016-0208-5 26861172 PMC4748603

[19] El-Menyar A, Sulaiman K, Almahmeed W, et al. Shock index in patients presenting with acute heart failure: A multicentre multinational observational study. Angiology. 2019;70(10):938–946. 10.1177/0003319719857560 31242749

[20] Borke J, Schraga ED, Schrediber D, et al. Natriuretic peptides in congestive heart failure. Medscape (WebMD LLC group) based in Newark, New Jersey, USA; 2019.

[21] Gounden V, Jialal I. Renal function tests. Stat pearls LLC based in Treasure Island, Florida, USA; 2019.

[22] Workeneh BT, Mamlouk O, Agraharkar M, et al. Acute Kidney Injury (AKI). New Jersey, USA: Medscape; 2024.

[23] Soman S, Aurora L. Type 3 cardiorenal syndrome. In: McCullough PA, Ronco C, editors. Textbook of Cardiorenal Medicine. Cham: Springer International Publishing; 2021. p. 95–110.

[24] Xing TJ, Clinical classification of liver failure: Consensus, contraindications and new recommendations. J Clin Gastroenterol Hepatol. 2017;1:2. 10.21767/25757733.10000016

[25] Evans L, Rhodes A, Alhazzani W, et al. Surviving sepsis campaign: International guidelines for management of sepsis and septic shock 2021. Intensive Care Med. 2021;47(11):1181–1247. 10.1007/s00134-021-06506-y 34599691 PMC8486643

[26] Barnes R, Clarke D, Farina Z, et al. Vital sign based shock scores are poor at triaging South African trauma patients. Am J Surg. 2018;216(2):235–239. 28859918 10.1016/j.amjsurg.2017.07.025

[27] Costa YC, Cáceres L, Mauro V, et al. Shock index, modified shock index, and age-adjusted shock index as predictors of in-hospital death in acute heart failure. Sub analysis of the ARGEN IC. Curr Probl Cardiol. 2022;47(10):101309. 10.1016/j.cpcardiol.2022.101309 35810845

[28] Oh GC, An S, Lee HY, et al. Modified reverse shock index predicts early outcomes of heart failure with reduced ejection fraction. ESC Heart Fail. 2022;9(5):3232–3240. 35775109 10.1002/ehf2.14031 PMC9715832

[29] Sotello D, Yang S, Nugent K. Comparison of the shock index, modified shock index, and age shock index in adult admissions to a tertiary hospital. Southwest Respir Crit Care Chron. 2019;7(28):18–23. 10.12746/swrccc.v7i28.539

[30] Torabi M, Mirafzal A, Rastegari A, et al. Association of triage time shock index, modified shock index, and age shock index with mortality in emergency severity index level 2 patients. Am J Emerg Med. 2016;34:63–68. 10.1016/j.ajem.2015.09.014 26602240

[31] Heidarpour M, Sourani Z, Vakhshoori M, et al. Prognostic utility of shock index and modified shock index on long-term mortality in acute decompensated heart failure. Persian registry of cardiovascular disease/heart failure (PROVE/HF) study. Acta Cardiol. 2022;78(2):217–226. 10.1080/00015385.2022.2030554 35098893

[32] Vakhshoori M, Bondariyan N, Sabouhi S, et al. Impact of shock index, modified SI, and age-derivative indices on acute heart failure prognosis; A systematic review and meta-analysis. PLoS One. 2024;19(12):e0314528. 10.1371/journal.pone.0314528 39700173 PMC11658625

[33] Nkoke C, Damasceno A, Edwards C, et al. Differences in socio-demographic and risk factor profile, clinical presentation, and outcomes between patients with and without RHD heart failure in Sub-Saharan Africa: Results from the THESUS-HF registry. Cardiovasc Diagn Ther. 2021;11(4):980–990. 10.21037/cdt-21-112 34527521 PMC8410489

[34] Heidenreich PA, Bozkurt B, Aguilar D, et al. 2022 AHA/ACC/HFSA Guideline for the Management of Heart Failure: A Report of the American College of Cardiology/American Heart Association Joint Committee on Clinical Practice Guidelines. Circulation. 2022;145(18):e895–e1032. 10.1161/CIR.0000000000001073 35363499

